# Adaptive mechanism between dynamical synchronization and epidemic behavior on complex networks

**DOI:** 10.1063/1.3622678

**Published:** 2011-08-12

**Authors:** Kezan Li, Xinchu Fu, Michael Small, Zhongjun Ma

**Affiliations:** 1 School of Mathematics and Computing Science, Guilin University of Electronic Technology, Guilin 541004, People’s Republic of China; 2 Department of Mathematics, Shanghai University, Shanghai 200444, People’s Republic of China; 3 Department of Electronic and Information Engineering, Hong Kong Polytechnic University, Hung Hom, Kowloon, Hong Kong

## Abstract

Many realistic epidemic networks display statistically synchronous behavior which we will refer to as *epidemic synchronization.* However, to the best of our knowledge, there has been no theoretical study of epidemic synchronization. In fact, in many cases, synchronization and epidemic behavior can arise simultaneously and interplay adaptively. In this paper, we first construct mathematical models of epidemic synchronization, based on traditional dynamical models on complex networks, by applying the adaptive mechanisms observed in real networks. Then, we study the relationship between the epidemic rate and synchronization stability of these models and, in particular, obtain the conditions of local and global stability for epidemic synchronization. Finally, we perform numerical analysis to verify our theoretical results. This work is the first to draw a theoretical bridge between epidemic transmission and synchronization dynamics and will be beneficial to the study of control and the analysis of the epidemics on complex networks.

Exploring the correlation between different dynamical behaviors which may appear in complex networks is an important and interesting pursuit on the way toward understanding them better. Among these different dynamical behaviors, synchronization and epidemic spreading on networks are closely related. For example, with the spread of an infective disease, people may reduce the frequency of inter-personal contact and take collective protective measures with increased regularity (for example, by washing hands frequently with clear water and soap, avoiding going to crowded place, and so on). This means that the spread of information between people during transmission of an epidemic disease can induce spontaneously collective risk-minimisation behaviour in spite of (or at least independently of) the actual disease pathology. Similar phenomena can also be found in many animal-borne infections. Hence, synchronization of individual’s behavior and their epidemic behavior on networks can occur simultaneously and interplay adaptively. However, very little theoretical work has been done to consider these two dynamical behaviors together. In this work, we address this deficit. We will investigate mathematically the correlation between the dynamical synchronization and the epidemic behavior on complex networks for us to understand them in depth.

## I. INTRODUCTION

It is well known that complex networks can accurately describe the topological structure of many dynamical systems in the real world. For example, the world wide web can be considered as a network of websites with hyperlinks. The Internet is formed by routers with physical connections. Similarly, the human brain can be also regarded as a system of neurons linked by synapses. Many other examples can be also found: the global economy, market, food cycle, metabolism, disease spreading, computer virus, energy diffusion, etc. Recently, research in the area of complex network has advanced significantly and will lead to a deeper study and more practical applications in future. Synchronization dynamics and epidemic dynamics are two of the main research fields in complex network science. In the last ten years, with the important discovery of small-world and scale-free networks, synchronization in complex networks has attracted great attention.^1–13^ Epidemic transmission in complex networks has also become an important research focus as it offers potential to advance the simple assumptions of homogeneously mixed models.^14–19^

Since synchronization and epidemic spreading are apparently two quite distinct behaviors, there is very little work to consider them together. Actually, synchronization and epidemic behavior on networks can arise simultaneously and interplay adaptively. Many realistic epidemic networks have displayed statistically synchronized behavior that we define as the epidemic synchronization which, for the moment, can be classified as *epidemic induced synchronization* and *epidemic characteristic synchronization*. For examples, with the spread of SARS (the severe acute respiratory syndrome), Bird Flu, or H1N1 influenza, people will spontaneously take some protective measures such as washing hands frequently with clear water and soap, avoiding going to crowded place, and so on in order to improve self-protection.^20^ When rhinoceroses and hippopotami are infected with epidemic ringworm, they will spontaneously go to a lakefront to have a bath in mud which will also provide either cure or protection. As the spread of these epidemic diseases becomes weak, these spontaneous protective behaviors (in either humans or animals) will decrease accordingly. Here, we choose to describe these consistent human or animal behaviors, which result from epidemic spreading, as the *epidemic induced synchronization*. For another example, weekly measles case reports^21^ for Birmingham, Newcastle, Cambridge, and Norwich between 1944 and 1958 have shown their singular oscillatory behavior and an in-phase synchronized pattern between Birmingham and Newcastle, but an anti-phase pattern between Cambridge and Norwich. Similarly, the reported cases of syphilis and gonorrhoea for the Midwest, South, Northeast, and West cities in United States between 1941 and 2002 have also exhibited the oscillatory characteristic and phase synchronized patterns.^22^ In addition, collective synchronization induced by epidemic dynamics on complex networks has also been studied numerically.^23^ For integrality, we define here these consistent oscillation of measures, which quantify the epidemic degree or other characteristics and do not refer to concrete human or animal behaviors, in epidemic networks as the *epidemic characteristic synchronization*. In this paper, we just address the epidemic induced synchronization.

The spatial synchronization of epidemics is considered to be a key factor affecting their long-term patterns of persistence and extinction.^24^ However, there are very few theoretical works exploring the real reason for epidemic synchronization apart from some numerical analyses from the view of statistics. So, it is significant to seek a reliable theory for epidemic synchronization. To this end, in this paper, we first establish the mathematical models to realize the epidemic synchronization and make further analysis of these models to reveal their exhibition mechanisms, based on the theory of dynamical systems.

This paper is organized as follows. In Sec. II, we introduce the traditional models of complex dynamical network and the epidemic network. In Sec. III, the models of SIS (the susceptible-infected-susceptible) epidemic synchronization and SIR (the susceptible-infected-recovered or the susceptible-infected-removed) epidemic synchronization are constructed. Then, we investigate the local and global conditions of epidemic synchronization with respect to the epidemic rate of these models. Finally, in Sec. IV, we use some numerical simulations to verify our theoretical results obtained in Sec. III. Section V concludes this paper and lists some possible further works.

## II. MODELS OF COMPLEX DYNAMICAL NETWORK AND EPIDEMIC NETWORK

Without loss of generality, we introduce a complex dynamical network^5^ with linear coupling which can be described as

$${\overset{\cdot }{x}}_{i}(t)=f(x_{i}(t))+c\sum_{j=1}^{N}a_{\mathrm{ij}}Hx_{j}(t),i=1,2,\ldots ,N,$$ (1)

where $x_{i}(t)\in R^{n}$ denotes the state variable of the *i*th node at the time *t*, and the function *f*(·) defines the local dynamics of each node and is supposed to be chaotic. The constant *c* > 0 is coupling strength and the matrix $H\in R^{n\times n}$ represents the inner-coupling matrix which is a constant 0 – 1 matrix linking coupled variables, and we assume it is positive. The coupling matrix *A* = (*a_ij_*)_*N* × *N*_ with zero-sum rows shows the coupling configuration of the network. If nodes *i* and *j* are connected, then *a_ij_* = *a_ji_* = 1, otherwise *a_ij_* = *a_ji_* = 0. The diagonal elements of the coupling matrix *A* are

$$a_{\mathrm{ii}}=-\sum_{j=1,j\neq i}^{N}a_{\mathrm{ij}}=-k_{i},i=1,2,\ldots ,N,$$ (2)

where *k_i_* denotes the degree of node *i*. With these assumptions, the eigenvalues of matrix *A* can be given by $0=\lambda_{1}\geq \lambda_{2}\geq \ldots \lambda_{N}$. Hence, by matrix theory, there exists unitary matrix *U* such that *A* = *U*Λ*U^T^*, where *U^T^U* = *I*, Λ = *diag*(*λ*_1_, *λ*_2_, … ,*λ_N_*).

Now, the standard SIS and SIR epidemic models^17,19^ in a complex network can be written as

$${\overset{\cdot }{I}}_{k}(t)=\lambda k[1-I_{k}(t)]\Theta (t,k)-I_{k}(t),k=1,2,\ldots ,d_{m}$$ (3)

and

$$\left\{ \begin{array}{c} {\overset{\cdot }{S}}_{k}(t)=-\lambda kS_{k}(t)\Theta (t,k),\\ {\overset{\cdot }{I}}_{k}(t)=\lambda kS_{k}(t)\Theta (t,k)-I_{k}(t),\\ {\overset{\cdot }{R}}_{k}(t)=I_{k}(t), \end{array}k=1,2,\ldots ,d_{m}\right.,$$ (4)

respectively. Here, the epidemic rate λ∈(0,1] denotes the probability with which each susceptible node is infected if it is connected to one infected node and *d_m_* represents the maximal degree in the network. The variables *S_k_*(*t*), *I_k_*(*t*), and *R_k_*(*t*) denote the densities of susceptible, infected, and removed nodes (individuals) with connectivity (contact) *k* at time *t*, respectively. These variables satisfy the normalization condition *S_k_*(*t*) + *I_k_*(*t*) = 1 or *S_k_*(*t*) + *I_k_*(*t*) + *R_k_*(*t*) = 1 for all *k*-classes in model (3) or (4). The term Θ(*t*,*k*) gives the probability that a randomly chosen link emanating from a node of connectivity *k* leads to an infected node. Moreover, Θ(*t*,*k*) has the form

$$\Theta (t,k)=\sum_{k'}p(k'|k)I_{k'}(t),$$ (5)

where the conditional probability p(k'|k) means that a randomly chosen link emanating from a node of connectivity *k* leads to a node of connectivity *k*′. We suppose that the connectivities of nodes in the whole network are uncorrelated, i.e., p(k'|k)=k'p(k')/〈k〉, where $〈k〉=\sum_{s}sp(s)$. The further details of models (3) and (4) can be found in Refs. 17 and 19.

## III. MODELS OF EPIDEMIC SYNCHRONIZATION AND ITS ANALYSIS

Based on the above traditional models (1) and (3), we can construct the following model of SIS epidemic synchronization:

$$\{\begin{array}{c} {\dot{x}}_{i}(t)=f(x_{i}(t))+c(t)\sum_{j=1}^{N}a_{ij}Hx_{j}(t),\\ {\dot{I}}_{k}(t)=\lambda k[1-I_{k}(t)]\Theta (t,k)-I_{k}(t),\\ \dot{c}(t)=\frac{\alpha I(t)}{N}\sum_{j=1}^{N}\frac{\|s(t)-{x_{j}(t)\|}^{2}}{1+\|s(t)-{x_{j}(t)\|}^{2}}, \end{array}$$ (6)

where *i* = 1, 2, …, *N*, *k* = 1, 2, … , *d_m_*, parameter *α* > 0, and $I(t)=\sum_{k=1}^{d_{m}}p(k)I_{k}(t)$. The initial condition of system (6) can be set as follows. The initial state *x_i_*(0) is chosen randomly from real number set and *I_k_*(0) = *ρ*, *c*(0) = 0 with 0<ρ≪1. The state variable *s*(*t*) is the synchronous state of system (6) with respect to epidemic induced synchronization or epidemic characteristic synchronization.

By the similar entrainment mechanism between the above traditional models (1) and (4), we can design the following model of SIR epidemic synchronization as

$$\{\begin{array}{l} {\dot{x}}_{i}(t)=f(x_{i}(t))+c(t)\sum_{j=1}^{N}a_{ij}Hx_{j}(t),\\ {\dot{S}}_{k}(t)=-\lambda kS_{k}(t)\Theta (t,k),\\ {\dot{I}}_{k}(t)=\lambda kS_{k}(t)\Theta (t,k)-I_{k}(t),\\ {\dot{R}}_{k}(t)=I_{k}(t),\\ \dot{c}(t)=\frac{\alpha I(t)}{N}\sum_{j=1}^{N}\frac{\|s(t)-{x_{j}(t)\|}^{2}}{1+\|s(t)-{x_{j}(t)\|}^{2}}, \end{array}$$ (7)

where *i* = 1, 2, … , *N*, *k* = 1, 2, … , *d_m_*, parameter *α* > 0, and epidemic prevalence $I(t)=\sum_{k=1}^{d_{m}}p(k)I_{k}(t)$.The initial condition of system (7) can be set as follows. The initial state *x_i_*(0) is chosen randomly from real number set and *S_k_*(0) = *ρ*, *I_k_*(0) = 1 – *ρ*, *R_k_*(0) = 0, *c*(0) = 0, where 0<ρ≪1.

When we consider the epidemic induced synchronization, *x_i_*(*t*) indicates the state variable of node-*i* in the epidemic network. In this case, the models (1) and (3) (similarly for Eqs. (1) and (4)) have the same topological structure, i.e., dynamical process of individuals and epidemic process arise simultaneously in the same network. And *f* defines the locally dynamical behavior which indicates the summation of various behavior of individual node concerning epidemic. By supposing that *f* is chaotic, we mean that the functional behaviour of each individual node is active, dynamic, and sufficiently complex.

When we take the epidemic characteristic synchronization into account, the variables *x_i_*(*t*) denotes the various factors influencing the epidemic transmission (such as the weekly infection rate of cities) and *f* shows the locally dynamical behavior of that variable. In this case, the models (1) and (3) (similarly for Eqs. (1) and (4)) may have different general topological structures. In this paper, we just address the first case where the synchronization is induced by the epidemic dynamics, which is realized by the adaptive coupling strength *c*(*t*) mainly depending on the change of *I*(*t*) and synchronization error.

In the third equation of model (6), the change of *c*(*t*) is controlled synthetically by *I*(*t*) and $\sum_{j=1}^{N}\frac{\|s(t)-{x_{j}(t)\|}^{2}}{1+\|s(t)-{x_{j}(t)\|}^{2}}$, where *I*(*t*) denotes the average density of infected nodes and this summation term can measure synchronization degree in a network. On one hand, individuals will send spontaneously safeguard information more frequently to protect themselves when disease prevalence becomes larger. So, it is reasonable to conclude that the rate of change of the coupling strength $\overset{\cdot }{c}(t)$ is directly proportional to the density *I*(*t*) of infected nodes. On the other hand, when the collective protective behavior increases sufficiently, communication of safeguard information among individuals will become stable since they have come to an agreement of protection. Thus, the proportional relation between $\overset{\cdot }{c}(t)$ and synchronization error $\sum_{j=1}^{N}\frac{\|s(t)-{x_{j}(t)\|}^{2}}{1+\|s(t)-{x_{j}(t)\|}^{2}}$ is also valid. We take the same consideration in the fourth equation of model (7).

### A. Local stability of epidemic synchronization

For the network system (1), we have the following basic result.

*Lemma 1*: Considering the network (1) with chaotic individual nodes, the coupling strength *c* = *c*(*t*), *H* = *I_N_*, and the maximal Lyapunov Exponent *h_max_* of function *f*, if there is *T* > 0 such that

$$c(t)>\frac{h_{\max}}{\left|\lambda_{2}\right|},\mathrm{for}t>T,$$ (8)

then the synchronization of network (1) is exponentially stable.

*Proof*: By reference,^25^ we know that the *n*(*N* – 1) Lyapunov exponents of network (1) on the synchronization manifold can be expressed as $\mu_{i}(\lambda_{k})=h_{i}+\lim\;\sup_{t\to \infty }c(t)\lambda_{k}$, where *h_i_* is the Lyapunov exponent of *f* and *i* = 1, 2, … , *n*, *k* = 2, 3, … , *N*. If there is *T* > 0 such that $c(t)>\frac{h_{\max}}{\left|\lambda_{2}\right|}$ for all *t* > *T*, then we get $h_{i}+\lim\;\sup_{t\to \infty }c(t)\lambda_{k}<0$ for all *i* = 1, 2, … , *n*, *k* = 2, 3, … , *N*. This means all *n*(*N* – 1) Lyapunov exponents of network (1) on the synchronization manifold are negative. According to the transverse stability of synchronization,^25^ we obtain the exponential stability of network (1).*□*

**Theorem 1:** Suppose that *λ_c_* > 0 is the epidemic threshold of system (3) and *f*(·) is a chaotic function of the corresponding model (6) with *H* = *I_N_*. If the epidemic rate *λ* > *λ_c_* in system (6), then *x_i_*(*t*), *i* = 1, 2, … ,*N* can achieve synchronization.

*Proof*: If *λ* > *λ_c_*, by the definition of epidemic threshold, there is a constant $I^{*}\in (0,1$] such that ${{\text{lim}}_{t}}_{\to +\infty }I\left(t\right)=I^{*}$. By defining $\delta (t)=\frac{\alpha I(t)}{N}\sum_{j=1}^{N}\frac{\|s(t)-{x_{j}(t)\|}^{2}}{1+\|s(t)-{x_{j}(t)\|}^{2}}$, we know *δ*(*t*) ≥ 0. Now, we show that *x_i_*(*t*), *i* = 1, 2, … ,*N* can realize synchronization in model (6).

If this is not the case, we obviously have ${\text{lim}}_{t\to +\infty }\delta \left(t\right)\neq 0$. Now, we will prove ${\text{lim}}_{t\to \infty }c\left(t\right)=+\infty$. In order to verify this limitation, we first show *c*(*t*) is boundless. Otherwise, there exists *M* > 0 such that |*c*(*t*)| < *M* for all *t* ≥ 0. By noting $\overset{\cdot }{c}(t)=\delta (t)\geq 0$, we can deduce that *c*(*t*) must have limitation with *t* → + *∞*, which means ${\text{lim}}_{t\to \infty }\delta \left(t\right)=0$. So *c*(*t*) is unbounded in (0, + *∞*). By combining the monotone property of *c*(*t*), we get ${\text{lim}}_{t\to \infty }c\left(t\right)=+\infty$. On the other hand, by Lemma 1, *x_i_*(*t*), *i* = 1, 2, … , *N* can achieve synchronization if $c\left(t\right)>h_{\max}/\left|\lambda_{2}\right|$. This contradiction concludes this theorem.*□*

**Theorem 2:** Suppose that *λ_c_* > 0 is the epidemic threshold of system (3) and *f*(·) is a chaotic function of the corresponding model (7) with *H* = *I_N_*. If the epidemic rate *λ* > *λ_c_* in system (7), then *x_i_*(*t*), *i* = 1, 2, … , *N* can achieve synchronization.

*Proof*: The proof is similar to the proof of theorem 1, we omit it here for simplicity.*□*

Now, we consider the infinite state of coupling strength *c*(*t*).

**Theorem 3:** Considering the model (6) or (7), for every λ∈(0,1), there is always a constant $c^{*}>0$, such that ${{\text{lim}}_{t}}_{\to \infty }c\left(t\right)=c^{*}$.

*Proof*: Obviously, for the model of epidemic synchronization (6) or (7), its epidemic threshold satisfies $\lambda_{c}\in \left(0,1\right)$ in its corresponding epidemic model. Now, we will prove this theorem for two cases with respect to *λ*.

On one hand, if 1 < *λ* ≤ *λ_c_*, by the above analysis, we know that *x_i_*(*t*), *i* = 1, 2, … ,*N* can realize synchronization. This means that ${{\text{lim}}_{t}}_{\to \infty }∥s\left(t\right)-x_{j}\left(t\right)∥=0$ for every j∈{1,2,…,N}. Moreover, it is easy to see that $0<\frac{\alpha I(t)}{N}\leq \frac{\alpha }{N}$. So, we get ${\text{lim}}_{t\to \infty }\overset{\cdot }{c}(t)=0$ for the model of SIS epidemic synchronization (6) or SIR epidemic synchronization (7). By combining the initial condition $c(0)=0,\overset{\cdot }{c}(0)>0$ and inequality $\overset{\cdot }{c}(t)\geq 0$, we find that there is always a constant c^*^ > 0, such that ${{\text{lim}}_{t}}_{\to \infty }c\left(t\right)=c^{*}$.

On the other hand, if 0 < *λ* ≤ *λ_c_*, we have ${{\text{lim}}_{t}}_{\to \infty }I\left(t\right)=0$ for the models (6) and (7) by the definition of epidemic threshold. In addition, it is obvious to see that

$$\frac{\alpha }{N}\sum_{j=1}^{N}\frac{\|s(t)-{x_{j}(t)\|}^{2}}{1+\|s(t)-{x_{j}(t)\|}^{2}}<\alpha .$$

Thus, for the model of SIS epidemic synchronization (6) or SIR epidemic synchronization (7), we obtain ${\text{lim}}_{t\to \infty }\overset{\cdot }{c}(t)=0$. In this case, by combining the initial condition c(0)=0, $\overset{\cdot }{c}(0)>0$, and inequality $\overset{\cdot }{c}(t)\geq 0$, we obtain the same result. Hence, the Theorem is proved.*□*

### B. Global stability of epidemic synchronization

Based on the paper,^10^ the synchronous state in network (6) or (7) can be defined as $s(t)=\frac{1}{N}\sum_{i=1}^{N}x_{i}(t)$. Hence, the corresponding synchronization errors can be set as *e_i_*(*t*) = *x_i_*(*t*) – *s*(*t*), *i* = 1, 2, … ,*N*. It is easy to obtain $\sum_{i=1}^{N}e_{i}(t)=0$ and $\overset{\cdot }{s}(t)=\frac{1}{N}\sum_{i=1}^{N}f(x_{i}(t))$. Consequently, the error system can be described as

$${\overset{\cdot }{e}}_{i}(t)=f(x_{i}(t))-f(s(t))+c(t)\sum_{j=1}^{N}a_{\mathrm{ij}}He_{j}(t)+g(t),i=1,2,\ldots ,N,$$ (9)

where $g(t)=f(s(t))-\frac{1}{N}\sum_{i=1}^{N}f(x_{i}(t))$.

**Hypothesis 1:** Suppose that *P* = *diag*(*p*_1_,*p*_2_, … ,*p_n_*) is a positive matrix. If there is a constant *ξ*, such that for all $x\left(t\right),y\left(t\right)\in R^{n}$, and *t* > 0, then we always have that

$${\left(x-y\right)}^{T}P[f(x)-f(y)]\leq \xi {\left(x-y\right)}^{T}(x-y).$$ (10)

By letting $F\left(t\right)={\left(f{\left(x_{1}\left(t\right)\right)}^{T}-f{\left(s\left(t\right)\right)}^{T},\ldots ,f{\left(x_{1}\left(t\right)\right)}^{T}-f{\left(s\left(t\right)\right)}^{T}\right)}^{T}$, $G\left(t\right)={\left(g^{T},\ldots ,g^{T}\right)}^{T}$, and $e\left(t\right)={\left(e_{1}^{T},\ldots ,e_{N}^{T}\right)}^{T}$, then the system (9) is rewritten as

$$\overset{\cdot }{e}(t)=F(t)+c(t)(A⊗H)e(t)+(I_{N}⊗I_{n})G(t),$$ (11)

where ⊗ is Kronecker product.

**Theorem 4:** Suppose that *λ_c_* > 0 is the epidemic threshold of system (3). If *λ* > *λ_c_* in system (6), then the synchronous manifold of system (6) is globally asymptotically stable.

*Proof*: We construct the following Lyapunov function candidate

$$V(t)=\frac{1}{2}e^{T}(t)(I_{N}⊗P)e(t)+\frac{1}{2}\beta {\left(c_{0}-c(t)\right)}^{2},$$

where *β* = − *λ*_2_*λ_min_*(*PH*) > 0, *λ_min_*(*PH*) denote the minimal eigenvalue of matrix *PH* and *c*_0_ is a undetermined constant. The derivative of *V*(*t*) with respect to *t* along the solution of system (6) is given by

$$\frac{\mathrm{dV}(t)}{\mathrm{dt}}=\sum_{i=1}^{N}e_{i}{\left(t\right)}^{T}P[f(x_{i}(t))-f(s(t))]+c{\left(t)e(t\right)}^{T}(A⊗\mathrm{PH})e(t)+e{\left(t\right)}^{T}(I_{N}⊗P)G(t)-\beta (c_{0}-c(t))\overset{\cdot }{c}(t)=\sum_{i=1}^{N}e_{i}{\left(t\right)}^{T}P[f(x_{i}(t))-f(s(t))]+c(t)e{\left(t\right)}^{T}(A⊗\mathrm{PH})e(t)-\beta (c_{0}-c(t))\overset{\cdot }{c}(t)\leq \xi \sum_{i=1}^{N}e_{i}{\left(t\right)}^{T}e_{i}(t)+c(t)e{\left(t\right)}^{T}(A⊗\mathrm{PH})e(t)-\beta (c_{0}-c(t))\overset{\cdot }{c}(t).$$ (12)

Now, introducing a transformation $y\left(t\right)={\left(y_{1}^{T}\left(t\right),\ldots ,y_{N}^{T}\left(t\right)\right)}^{T}=\left(U^{T}⊗I_{n}\right)e\left(t\right)$ and combining Eq. (12), we obtain

$$\frac{\mathrm{dV}(t)}{\mathrm{dt}}\leq \xi \sum_{i=1}^{N}e_{i}{\left(t\right)}^{T}e_{i}(t)+c(t)y^{T}(t)(\Lambda ⊗\mathrm{PH})y(t)-\beta (c_{0}-c(t))\overset{\cdot }{c}(t)=\xi \sum_{i=1}^{N}e_{i}{\left(t\right)}^{T}e_{i}(t)-\beta c_{0}\overset{\cdot }{c}(t)+c(t)y^{T}(t)(\Lambda ⊗\mathrm{PH})y(t)+\beta c(t)\overset{\cdot }{c}(t)\leq \xi \sum_{i=1}^{N}e_{i}{\left(t\right)}^{T}e_{i}(t)+c_{0}\lambda_{2}\lambda_{\min}(\mathrm{PH})\frac{\alpha I(t)}{N}\sum_{i=1}^{N}\frac{e_{i}{\left(t\right)}^{T}e_{i}(t)}{1+e_{i}{\left(t\right)}^{T}e_{i}(t)}+c(t)\lambda_{2}\lambda_{\min}(\mathrm{PH})\frac{\alpha I(t)}{N}\sum_{i=1}^{N}\frac{e_{i}{\left(t\right)}^{T}e_{i}(t)}{1+e_{i}{\left(t\right)}^{T}e_{i}(t)}-c(t)\lambda_{2}\lambda_{\min}(\mathrm{PH})\overset{\cdot }{c}(t).$$ (13)

If epidemic rate *λ* > *λ_c_*, then there exists $I^{*}\in \left(0,1\right]$, such that ${{\text{lim}}_{t}}_{\to +\infty }I\left(t\right)=I^{*}$. By choosing ${ɛ}_{0}\in \left(0,I^{*}\right)$, then there is *t*_0_ such that $I\left(t\right)>I^{*}-{ɛ}_{0}>0$ for all *t* > *t*_0_. When *t* > *t*_0_, by Eq. (13) we have

$$\frac{\mathrm{dV}(t)}{\mathrm{dt}}\leq \xi \sum_{i=1}^{N}e_{i}{\left(t\right)}^{T}e_{i}(t)+c_{0}\lambda_{2}\lambda_{\min}(\mathrm{PH})\frac{\alpha (I^{*}-{ɛ}_{0})}{N}\sum_{i=1}^{N}\frac{e_{i}{\left(t\right)}^{T}e_{i}(t)}{1+e_{i}{\left(t\right)}^{T}e_{i}(t)}+c(t)\lambda_{2}\lambda_{\min}(\mathrm{PH})\left[\frac{\alpha I(t)}{N}\sum_{i=1}^{N}\frac{e_{i}{\left(t\right)}^{T}e_{i}(t)}{1+e_{i}{\left(t\right)}^{T}e_{i}(t)}-\overset{\cdot }{c}(t)\right]=\xi \sum_{i=1}^{N}e_{i}{\left(t\right)}^{T}e_{i}(t)+c_{0}\frac{\alpha \lambda_{2}\lambda_{\min}(\mathrm{PH})(I^{*}-{ɛ}_{0})}{N}\sum_{i=1}^{N}\frac{e_{i}{\left(t\right)}^{T}e_{i}(t)}{1+e_{i}{\left(t\right)}^{T}e_{i}(t)}.$$

Thus, we can select an adequately large constant *c*_0,_ such that $\frac{\mathrm{dV}(t)}{\mathrm{dt}}\leq 0$. On the other hand, it is easy to see that $\frac{\lambda_{\min}(P)}{2}e^{T}(t)e(t)+\frac{1}{2}\beta {\left(c_{0}-c(t)\right)}^{2}\leq V(t)\leq \frac{\lambda_{\max}(P)}{2}e^{T}(t)e(t)+\frac{1}{2}\beta {\left(c_{0}-c(t)\right)}^{2}$. So, the Lyapunov function *V*(*t*) has infinitesimal upper bound and is infinitely large.^26,27^ Therefore, the zero solution of error system (9) is globally asymptotically stable, i.e., the synchronisation manifold of system (6) is globally asymptotically stable.*□*

Similarly, if epidemic rate *λ* > *λ_c_*, the synchronisation manifold of system (7) is also globally asymptotically stable.

*Remark 1*: Actually, we just need the existence of the above constant *ξ* in Hypothesis 1 to achieve the global stability of epidemic synchronization, as shown by the above analysis. So, it is convenient to use Theorem 4 for analysis in realistic applications.

*Remark 2:.* In fact, since some certain synchronization patterns can accelerate or weaken epidemic prevalence, it is more realistic to consider the effect of these synchronization dynamics on epidemics synchronously, as we investigate the relationship between the epidemic dynamics and synchronization dynamics on a network. If we embed this anti-effect function in model (6) or (7), it must influence the epidemic process, i.e., either enhancing or reducing the epidemic prevalence *I*(*t*). But from the results obtained above, our synchronization conditions are all independent on this epidemic process, but depend only on the epidemic rate. So, the above synchronization conditions are still valid in this case.

## IV. NUMERICAL SIMULATION

**FIG. 1.**
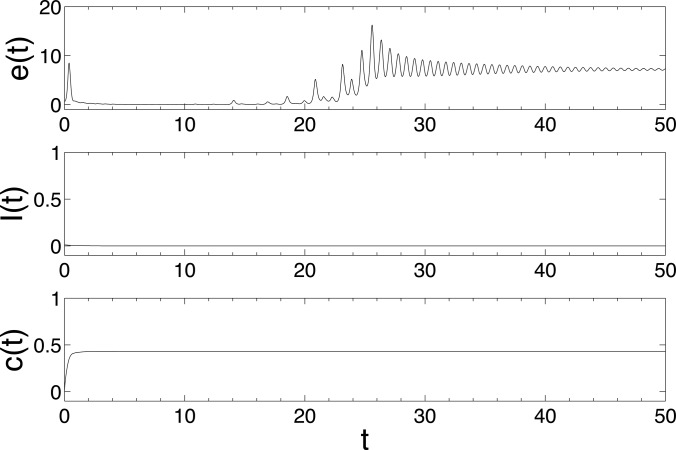
The changes of synchronization error *e*(*t*), epidemic prevalence *I*(*t*), and coupling strength *c*(*t*) in model (6) with epidemic rate *λ* = 0.02. The network is BA scale-free network with size *N* = 500, the maximal degree *d_m_* = 60 and 192 nodes with the minimal degree 3. Other parameters are set as *α* = *N* – 1, initial infection density *ρ*_3_ = 0.03125, *ρ_k_* = 0, *k* = 4,…,*d_m_*, i.e., there are originally 6 infected nodes with degree 3 and the other nodes are all susceptible.

**FIG. 2.**
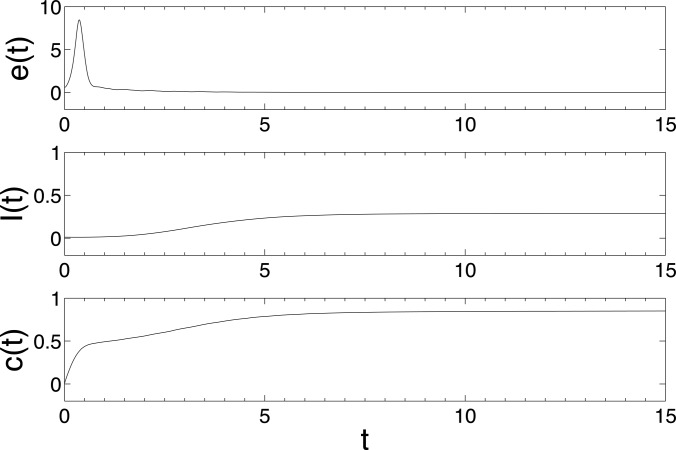
The changes of synchronization error *e*(*t*), epidemic prevalence *I*(*t*), and coupling strength *c*(*t*) in model (6) with epidemic rate *λ* = 0.1. The network is BA scale-free network with size *N* = 500, the maximal degree *d_m_* = 60 and 192 nodes with the minimal degree 3. Other parameters are set as *α* = *N* – 1, initial infection density *ρ*_3_ = 0.03125, *ρ_k_* = 0, *k* = 4,…,*d_m_*, i.e., there are originally 6 infected nodes with degree 3 and the other nodes are all susceptible.

**FIG. 3.**
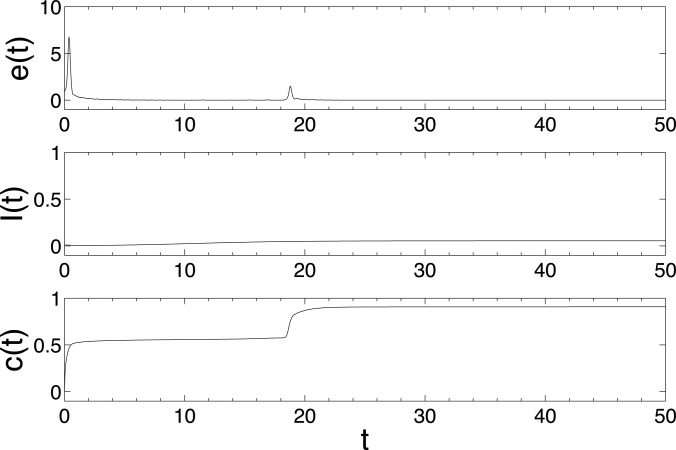
The changes of synchronization error *e*(*t*), epidemic prevalence *I*(*t*), and coupling strength *c*(*t*) in model (6) with epidemic rate *λ* = 0.2. The network is BA scale-free network with size *N* = 500, the maximal degree *d_m_* = 60 and 192 nodes with the minimal degree 3. Other parameters are set as *α* = *N* – 1, initial infection density *ρ*_3_ = 0.03125, *ρ_k_* = 0, *k* = 4,…,*d_m_*, i.e., there are originally 6 infected nodes with degree 3 and the other nodes are all susceptible.

To verify the above results, we now investigate numerically the model of SIS epidemic synchronization (6). Since this analysis can be generalized analogously to the model of SIR epidemic synchronization (7), we omit this generalization for simplicity. The network embedded in model (6) is made to be the BA (the Barabasi and Albert model) scale-free (preferential attachment) network^28^ with size *N* = 500. This network evolves from initial network with size *m*_0_ = 4 and we add each new node with *m* = 3 new edges. Without loss of generality, we suppose that the *f* in model (6) is defined as the chaotic Lorenz oscillation. Certainly, this assumption is rather difficult to justify from the point of view of a physical epidemic transmission process, but we just use it for numerical simulations. This oscillation can be described as

$$\left\{ \begin{array}{c} \frac{dx_{1}(t)}{\mathrm{dt}}=a_{1}(x_{2}(t)-x_{1}(t))\\ \frac{dx_{2}(t)}{\mathrm{dt}}=a_{2}x_{1}(t)-x_{2}(t)-x_{1}(t)x_{3}(t)\\ \frac{dx_{3}(t)}{\mathrm{dt}}=x_{1}(t)x_{2}(t)-a_{3}x_{3}(t) \end{array}\right.$$ (14)

where parameters *a*_1_ = 10, *a*_2_ = 28, and *a*_3_ = (8/3). *H* is chosen as the identity matrix. The Figures 1, 2, and 3 show the changes in synchronization error *e*(*t*), epidemic prevalence *I*(*t*), and coupling strength *c*(*t*) in the model (6) with epidemic rate *λ* = 0.02, 0.1, and 0.2, respectively. Here, the synchronization error is defined as $e(t)=\sum_{j=2}^{N}\left\|x_{1}(t\right)-x_{j}(t)\|/N$. From Fig 1, we can see that *I*(*t*) → 0(*t* → *∞*), i.e., the epidemic does not break out. In this case, the synchronization error *e*(*t*) does not converge to zero, which means that the epidemic dynamics cannot successfully induce the synchronization of individuals under small epidemic rate *λ* = 0.02. From Fig 2, we can see that *I*(*t*) converges to a positive number and *e*(*t*) converges to zero, which implies that the epidemic dynamics can induce successfully the synchronization of individuals with larger epidemic rate *λ* = 0.1. Increasing further the epidemic rate to 0.2, we find that the epidemic dynamics can not only induce successfully the synchronization, but also enhance the speed of synchronization. These simulations show that if the epidemic transmission spreads more easily on the network, then the epidemic dynamics can induce the synchronization of individuals more effectively. This is consistent with the attained theoretic results in Sec. III—*an aggressive disease leads to a strong response from individuals.*

## V. CONCLUSIONS

Based on the synchronization behavior of individuals induced by epidemic dynamics in real epidemic networks, this paper has proposed some mathematical models of epidemic synchronization which can characterize this kind of phenomenon very well. We have studied the relationship between the epidemic rate and synchronization stability of these models and obtained a very explicit condition for synchronization with respect to the epidemic rate, i.e., *λ* > *λ_c_*. Numerical simulations show that if the epidemic disease breaks out more easily, then the epidemic dynamics can induce the synchronization of its individuals more effectively. This conclusion accords very well with the characteristics of real epidemic networks.

In future, we will extend our work to address the following two issues. First, since in realistic epidemic networks the individuals are generally different, the local dynamics of nodes can not be identical in a whole network. So, it must be more appropriate to consider network models with both community structure and (distinct) individual behavior. Second, we anticipate that some certain synchronization patterns can accelerate or weaken epidemic prevalence. So, it may be very significant to consider the effect of these synchronization dynamics on epidemics when we study the epidemic synchronization on complex networks.
